# Crystallisation of 1,2-dimethylimidazole, and 1-methylimidazole with organic acids

**DOI:** 10.1038/s41598-025-34293-9

**Published:** 2026-01-05

**Authors:** Hadeer Q. Waleed, Rehana Bano, Anikó Csábrádiné Jordán, Olga Książkiewicz, Marcin Palusiak, Béla Fiser

**Affiliations:** 1 Department of Computer Engineering Techniques, College of Engineering Techniques, University of Bilad Alrafidain, Diyala, Iraq; 2 Higher Education and Industrial Cooperation Centre, University of Miskolc, Miskolc-Egyetemváros, 3515 Hungary; 3 BioMedChem Doctoral School of University of Łódz and Łódz Institutes of the Polish Academy of Sciences, Matejki 21/23, Łódz, 90-237 Poland; 4 Department of Physical Chemistry, Faculty of Chemistry, University of Łódz, Pomorska 163/165, Łódz, 91-236 Poland; 5 Institute of Chemistry, University of Miskolc, Miskolc-Egyetemváros, 3515 Hungary; 6 Ferenc Rakoczi II Transcarpathian Hungarian College of Higher Education, Beregszász, Transcarpathia, 90200 Ukraine

**Keywords:** Crystal structure, Hydrogen bond, Salt, Imidazole, Organic acids, Theoretical studies, Biochemistry, Chemistry

## Abstract

Crystals of 1-methylimidazole and 1,2-dimethylimidazole with glutaric and succinic acids are described. The crystal structure including 1,2-dimethylimidazole and glutaric acid, has been reported previously, but the molecular salt of 1-methylimidazole with succinic acid were characterized for the first time in this study. The structures were characterised by using X-ray crystallography, intermolecular hydrogen bond geometry, energy, and topology. In both crystal structures the molecules are linked by N-H….O and C-H….O hydrogen bonds. Using Hirshfeld surface analyses and quantum-theoretical calculations, we provide insights into the intermolecular interactions, revealing dependencies on the chemical environment. The result of the interaction region indicator (IRI) analysis indicated that succinic acid and 1-methylimidazole (II) exhibits low gradient, low density, and sharp spikes, whereas the spikes of the molecular salt of glutaric acid and 1,2-dimethylimidazole (I) are less dense. The quantum theory of atoms in molecules (QTAIM) analysis indicates that the described molecular salts exhibit different types of bond critical paths. These observations confirm the hydrogen bonded nature of molecular salts based on the characteristics of the bond critical points.

**Supplementary Information:**

The online version contains supplementary material available at 10.1038/s41598-025-34293-9.

## Introduction

The term “crystal engineering” was first introduced by R. Pepinsky in 1955. In the 1980s, Schmidt advanced the field through his remarkable work on topochemical reactions. Since then, crystal engineering has continued to attract growing interest from various disciplines, including solid-state chemistry, crystallography, inorganic chemistry, and theoretical chemistry^1–4^. The concept of crystal engineering has significantly contributed to the design of predicted cocrystals with desired physicochemical properties^5,6^. The synthesis method plays a critical role in determining the final crystalline product by influencing both kinetic and thermodynamic parameters. These factors can drive the formation of different polymorphic forms, modify the intended stoichiometry, or alter the resulting hydrogen-bonding networks^7^. Solvent selection and concentration exert a significant effect on crystal habit, as solvent molecules can interact with solute species in solution and promote specific molecular arrangements or synthons that become fixed during crystallization. Additionally, solution concentration governs the level of supersaturation, which in turn determines whether a thermodynamically stable phase or a metastable intermediate is favored^8,9^. Temperature further influences solubility and the position of the saturation point, while agitation (such as stirring intensity) affects nucleation and growth kinetics. Variations in agitation can even direct the system toward alternative polymorphic outcomes; for instance, higher stirring rates may promote the formation of a polymorph not obtained under static or low-agitation conditions^10^.

The crystallization process is fundamentally a balance between thermodynamics, which dictates what is possible (the most stable form), and kinetics, which dictates what actually happens within a given time frame (the rate of formation and the specific pathway taken). The crystallization process is primarily dictated by the thermodynamic characteristics of the system. Parameters such as solubility, supersaturation, and the extent of the metastable zone significantly influence subsequent kinetic behavior. Solubility serves as the foundation for crystallization design, determining expected yield and throughput, guiding solvent selection, and shaping the overall crystallization strategy. Equally important is the understanding of the metastable zone, which represents conditions where the solution remains supersaturated but does not yet undergo spontaneous nucleation. Operating within this region enables controlled crystal growth while preventing premature nucleation. Collectively, these thermodynamic factors are essential for achieving consistent, high-quality crystallization outcomes^11^. Meanwhile, the key kinetic parameters governing crystallization are the nucleation rate describing how quickly stable crystal nuclei form in a supersaturated solution and the growth rate, which reflects the speed at which these nuclei develop into larger, measurable crystals. Both parameters are commonly modeled as functions of operational variables such as supersaturation and temperature, and are characterized using defined constants and exponents^12^.

Organic salts formed from imidazoles and dicarboxylic acids in their anhydrous state are increasingly studied for their promise as pure organic proton-conducting electrolytes, particularly in fuel cells, which offer efficient energy conversion with minimal emissions^13^. Recently, manual mechanochemical grinding was used to synthesize phase-pure imidazole-based organic salts, which showed distinct electrical conductivities and activation energies compared to solution-grown crystals due to structural defects. The method enables full deuteration, tunable conductivity, and offers strong potential for large-scale industrial production^14^. While Impedance spectroscopy revealed that the electrical conductivity of five imidazolium dicarboxylate salts increases with temperature, with results linked to differences in crystal structure^15^.

As a result, controlling or predicting crystal structures remains a considerable challenge. The rational design of crystals typically relies on supramolecular synthons. When molecules can associate through multiple, potentially competing synthons, the design strategy must consider the hierarchy of these synthons that is, which synthons are preferentially formed over others^16,17^. Anhydrous organic salts composed of imidazoles and dicarboxylic acids have attracted significant attention for their potential use as pure organic proton-conducting electrolytes in fuel cells, a high-efficiency and low-emission energy conversion device^18^.

The term imidazole was first introduced by German chemist Arthur Rudolf Hantzsch in 1887^19^. Imidazole belongs to the class of heterocyclic compounds. It is highly soluble in water, has an electric dipole moment of 3.67D, and is highly polar^20^. Imidazole is classified as an amphoteric compound, acting as both an acid and a base. The compound is classified as aromatic due to the presence of a sextet of π electrons, consisting of a pair of nonbonding electrons from the nitrogen N atom and one from each of the four remaining ring atoms. Imidazole can form stable crystalline salts with strong acids through the protonation of the sp^2^ nitrogen (N), known as imidazolium salts. Imidazole has a pK_a_H of 7.1, acting as a strong base^21^. Among the different heterocyclic compounds, imidazole is better known due to its wide range of chemical and biological properties. It has become an important synthon in the development of new drugs^22^. Imidazole has a structure that combines the structural characteristics of pyrrole and pyridine. Three carbon atoms and the pyridine-type nitrogen each donate one electron to the aromatic sextet, whereas the pyrrole-type nitrogen contributes two electrons^23^. In addition, the imidazole group was proven effective as a catalyst for polyurethane, especially 1-methylimidazole and 1,2-dimethylimidazole^24,25^.

In an earlier study, researchers presented the findings of a systematic investigation into the solid-form products resulting from the crystallization of dicarboxylic acids (diacids) with the weakly basic cyclic amides 2-imidazolidinone and 2-pyrrolidinone^26^. Another study reported nineteen new crystal structures containing α,w-alkanedicarboxylic acids, maleic acid, and fumaric acid with imidazole, which were characterized by single-crystal X-ray diffraction^27^. To contribute to this area, we have succeeded in preparing two single-crystal structures. The first, (molecular salt I), 1,2-dimethylimidazole in complex with glutaric acid, has been reported previously, but 1-methylimidazole in complex with succinic acid (molecular salt II) in the crystalline state, was characterized for the first time by X-ray crystallography.

**Fig. 1:**
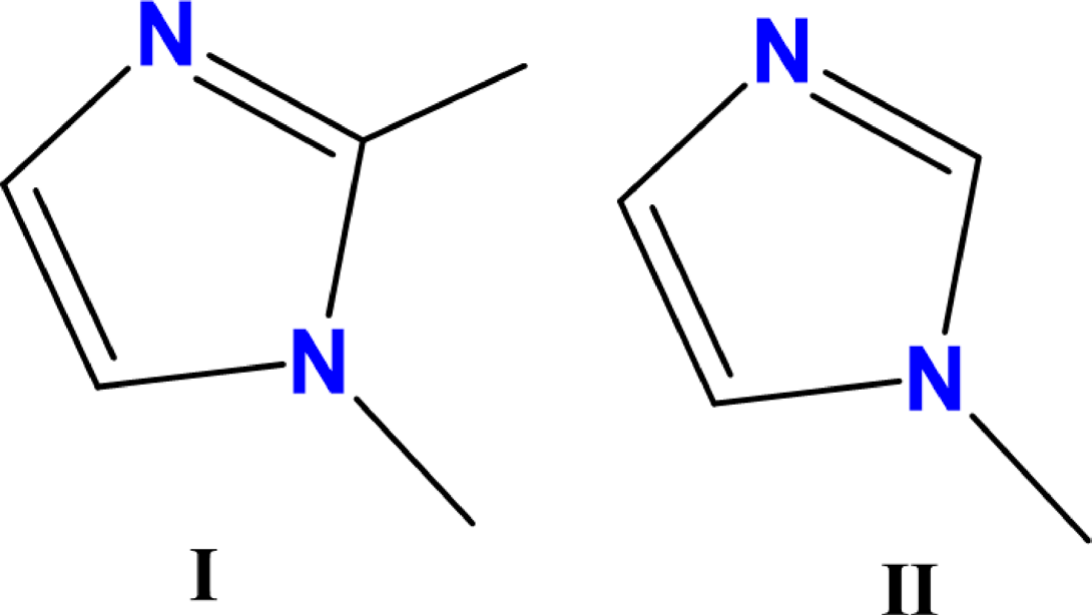
The molecular structure of (**I**) 1,2-dimethylimidazole, and (**II**) 1-methylimidazole.

In this paper, we present X-ray structural analyses of two structures involving 1-methylimidazole and 1,2-dimethylimidazole (Fig. 1), focusing on intermolecular hydrogen bonds, geometry, energy, and topology. Using Hirshfeld surface analyses and quantum-theoretical calculations, we provide insights into the intermolecular interactions, revealing dependencies on the chemical environment.

## Experimental

### Preparation of crystals

The crystals in this study were obtained from commercially available reagents (Sigma–Aldrich Chemical Co.) and used without further purification.

#### Preparation of (**I)**

First, 1,2-dimethylimidazole (0.0048 g) and glutaric acid (0.0066 g) were weighed using an analytical balance. The substances were combined in a 1:1 ratio and dissolved in 4 ml of ethanol. The solution was transferred to a 10 ml bottle and stirred with a magnetic stirrer for 3 min. Using a syringe with a filter, the solution was transferred to a crystallizing dish with a capacity of 20 ml. The crystallization was conducted in natural light, the crystallizing dish was sealed with Parafilma and left undisturbed in a controlled environment for two weeks.

#### Preparation of **(II)**

First, 1-methylimidazole (0.0041 g) and succinic acid (0.0059 g) were weighed using an analytical balance. The substances were combined in a 1:1 ratio and dissolved in 8 ml of ethanol and 1 ml of water. The solution was transferred to a 10 ml bottle and stirred with a magnetic stirrer for 3 min. Using a syringe with a filter, the solution was transferred to a crystallizing dish with a capacity of 20 ml. The crystallization was conducted in natural light, the crystallizing dish was sealed with Parafilma and left undisturbed in a controlled environment for two weeks.

### X-ray structure determination and refinement

The crystal structures’ data, and the corresponding data collection and structure refinement details are presented in Table 1.

**Table 1:** Experimental details. Experiments were carried out at room temperature with Cu *Kα* radiation (λ = 1.54184Å) using a Rigaku XtaLAB synergy dualflex diffractometer with a hypix detector.

Crystal data	(I)	(II)
Chemical formula	C_10_H_16_N_2_O_4_	C_16_H_20_N_4_O_8_*
Formula weight	228.25	396.36
Temperature/ K	293(2)	293(2)
Radiation	Cu Kα (λ = 1.54184Å)	Cu Kα (λ = 1.54184Å)
Crystal system, space group	orthorhombic, Pna2_1_	triclinic, P$\:\stackrel{-}{1}$
a, b, c (Å)	10.23300(10), 23.0144(3), 4.79660(10)	6.45150(10), 8.8082(2), 9.5671(2)
α, β, γ (º)	90, 90, 90	67.162(2), 75.309(2), 84.972(2)
V (Å^3^)	1129.63(3)	484.626(19)
Z	4	1
µ (mm^-1^)	0.875	0.944
Crystal size (mm)	0.569 × 0.060 × 0.043	0.648 × 0.410 × 0.210
Reflections collected	10,269	16,277
Idependent reflections	2070 [ R_int_= 0.0440, R_sigma_= 0.0301]	1903 [ R_int_= 0.02552, R_sigma_= 0.0125]
Data/restraints/parameters	2070/1/149	1903/0/132
Goodness-of-fit on F^2^	1.068	1.119
Final R indexes [I > = 2σ(I)]	R_1_ = 0.0343, wR_2_ = 0.0940	R_1_ = 0.0447, wR_2_ = 0.1325
Final R indexes [all data]	R_1_ = 0.0355, wR_2_ = 0.0952	R_1_ = 0.0515 wR_2_ = 0.1421
Δρ_max_, Δρ_min_ (e Å^-3^)	0.15/-0.12	0.26/-0.18
Number CCDC	2408255	2408256

**Fig. 2:**
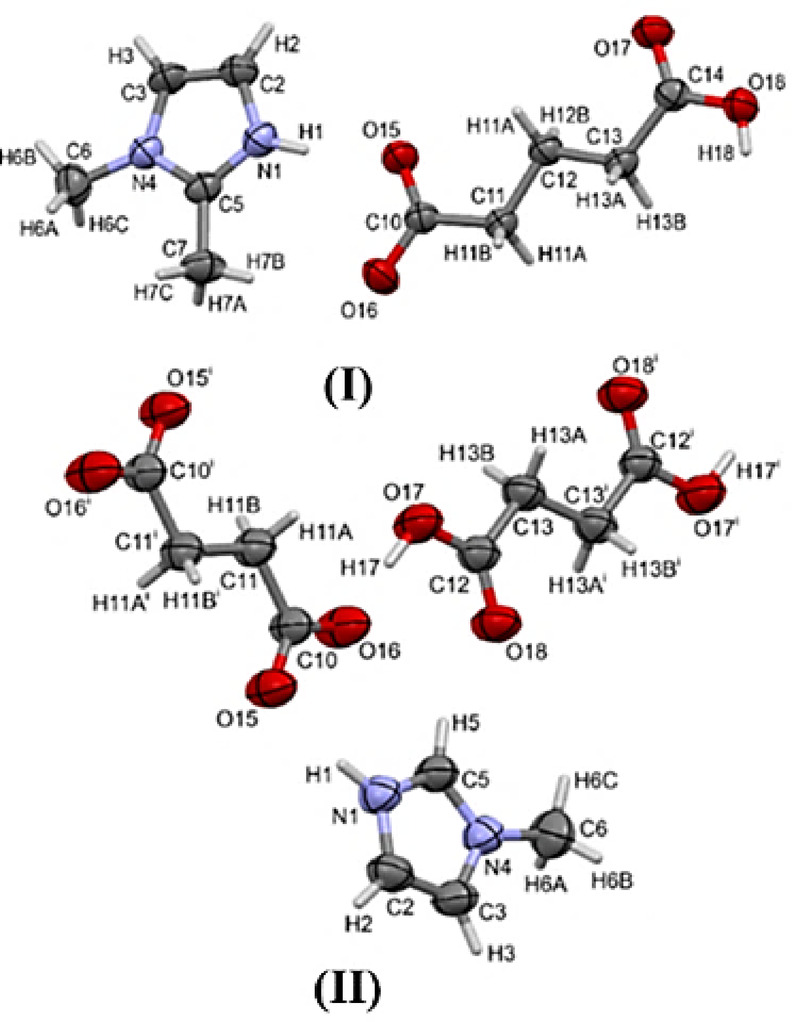
The molecular components of **(I)** & **(II)**, with the atom labeling schemes (from X-ray data).

* In case of the crystal of (II) there is 1:1 stoichiometry between 1-methylimidazole and dicarboxylic acid components, however, the acid molecules exist within the crystal in two forms, fully neutral one and dianionic one. What is more, in both cases the molecules are in special position (inversion at the center). Using this symmetry one gets the visualisation as in Fig. 2, molecular salt II. In this visualisation both dicarboxylic fragments (half of each molecule) are shown with symmetry equivalent counterparts (the counterpart, that is, second half of each molecular fragment).

### Hirshfeld surface analysis

Molecular Hirshfeld surfaces and fingerprint plots were generated using Crystal Explorer software^28^ through its automated procedures. The surfaces were mapped based on normalized contact distances (dnorm), with values ranging from − 0.8247 to 1.1694 a.u. for molecular salt **(I)** and from − 0.8481 to 1.1665 a.u. for molecular salt **(II)**.

### Theoretical calculations

In order to gain an understanding of the reactivity of the investigated compound, single point energy calculations were carried out on the structures using the B3LYP/6-31G(d, p) level of theory as implemented in the Gaussian 16 Programm Package^29^. The visualization of the geometries was carried out by using the GaussView 6.0 software^30^. The interaction region indicator (IRI), quantum theory of atom in molecules (QTAIM), and frontier molecular orbitals (FMOs) analyses have been carried out at B3LYP/6-31G(d, p) level of theory, and their electron densities and other parameters are obtained by Multiwfn^31^ and visual molecular dynamics (VMD) software^32^.

## Results and discussion

### Crystal structures and optimized geometries

A search of the CSD was conducted for crystal structures that include 1-methylimidazole and 1,2-dimethylimidazole, using molecular structure as the sole criterion. In our newly formed **(I)**, all components crystallize together in a 1:1 stoichiometric ratio. Compound (**I**) can be clearly classified as a salt. However, structure (**II**) contains one acid anion and one acid neutral molecule in the asymmetric unit. So, according to^33^ this structure fulfils criteria of both the salt and the cocrystal. The more universal qualification to describe both the structure (**I**) and the structure (**II**) with one name is the term “multicomponent crystal”. We use this term in case we are referring to both structures in the same time. In the case of structure (I) and (II) we do not observe the phase transition in crystalline state. Both (I) and (II) are stable after crystallization and could be used for X-ray experiments without any additional special treatment. The molecular structures are depicted with atoms represented by displacement ellipsoids (Fig. 2). The two structures contain chains of hydrogen bonded anion/ dianion-acid molecules that are further hydrogen bonded across the cations. In **(I)**, one of the methyl groups points toward the carboxyl group while the other methyl group points in the opposite direction, and in **(II)**, the methyl group point in the opposite direction from the carboxyl group (Fig. 2). Crystal components of (**I**) and (**II**) are shown in Fig. 2. In case of (**I**) this is the asymmetric unit. In case of (**II**) the asymmetric unit would contain half of succinic acid anion and half succinic acid neutral molecule. We show the entire molecules for clarity. The crystal ratio of (**II**) is 1:1/2:1/2 for 1-methylimidazole, succinic acid anion and succinic acid neutral molecule, respectively^33^.

**Fig. 3:**
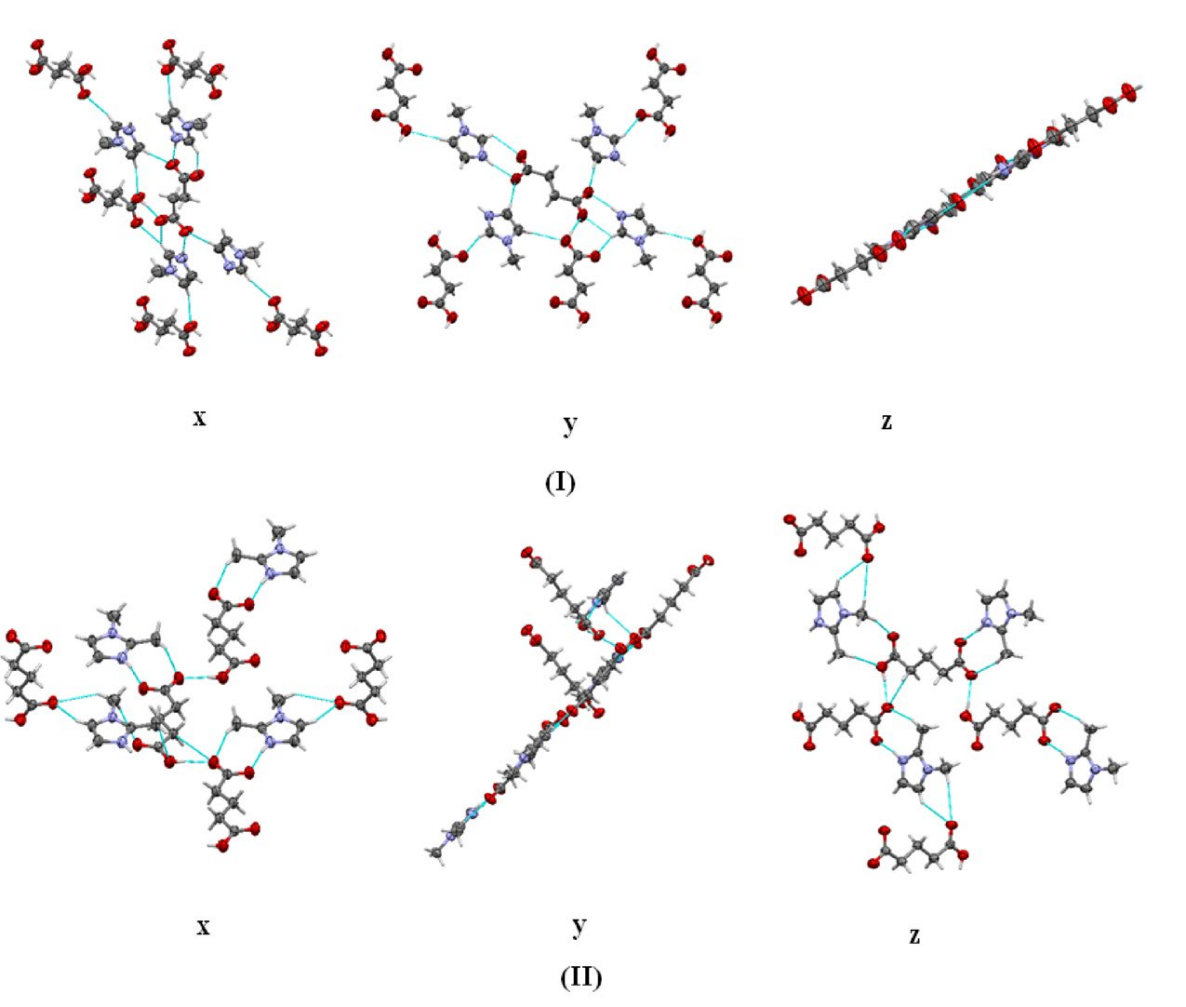
The hydrogen-bonding schemes along with the symmetry presented as blue dotted lines, in the (**I)** & (**II)**.

In both salts, the N—H….O and C—H….O hydrogen bonds are observed. The N1-H1 bond lengths (0.86 Å) are shorter than the H….O bond lengths in both molecular salts (Table 2**)**, which can be attributed to proton transfer and the presence of electron density near the N atom. The schemes of these interactions and their geometric parameters are presented in (Fig. 3; Table 2).

**Table 2:** Hydrogen-bond geometry (Å) for (**I)** & (**II)** with symmetries.

D—H…A	D—H	H…A	D…A	D—H…A	Symmetry codes
Molecular Salt I					
N1—H1…O15	0.86	1.86	2.7103(18)	172	x, y, z
N1—H1…O16	0.86	2.50	2.973(2)	115	x, y, z
O17—H17…O16	0.95(3)	1.55(3)	2.502(2)	178(4)	x, y, z
C2—H2…O15	0.93	2.25	3.173(2)	174	-x, 1-y, 1-z
C3—H3…O17	0.93	2.37	3.283(2)	167	x, y, 1 + z
C5—H5…O16	0.93	2.42	2.946(2)	116	x, y, z
C5—H5.O18	0.93	2.31	3.194(2)	159	x, y, z
C6—H6A…O16	0.96	2.55	3.501(3)	169	1-x,-y, 1-z
**Molecular Salt II**					
N1—H1…O15	0.86	1.81	2.659(3)	170	-½+x, ½-y, 1 + z
O18—H18…O16	0.82	1.71	2.530(2)	173	-½+x, ½-y, 1 + z
C3—H3.O17	0.93	2.47	3.268(3)	143	½-x, -½+y, -½+z
C6—H6A…O17	0.93	2.58	3.444(4)	149	½+x, ½-y, z
C7—H7B.O16	0.96	2.43	3.358(4)	162	-½+x, ½-y, 1 + z

**Fig. 4:**
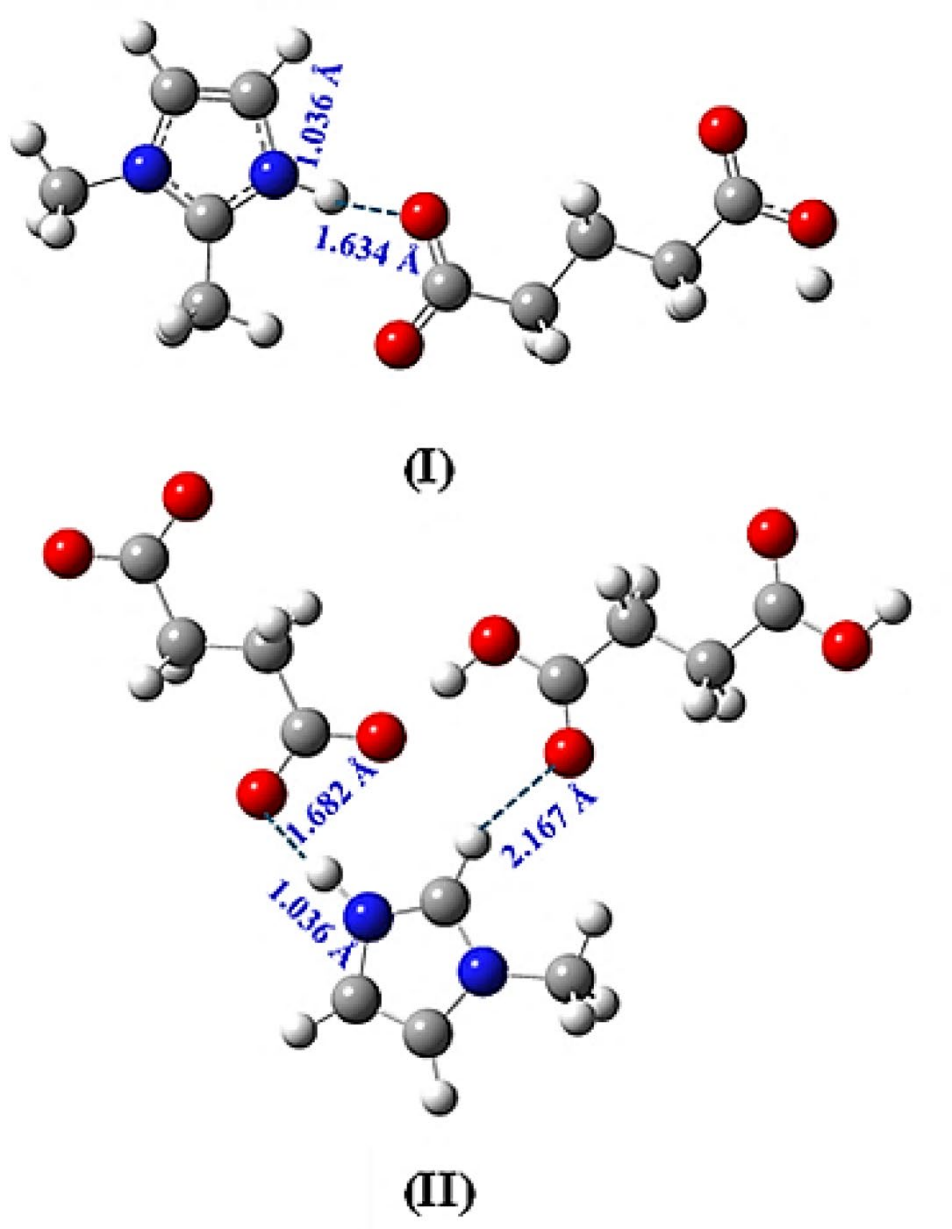
Graphical representations of **(I)** and **(II)**, showing the scaled bond lengths.

Furthermore, the X-H bonds of the X-ray crystallographic structures were scaled using neutron diffraction data reported previously to obtain accurate bond lengths^34^. After scaling, the single-point energy structures of **(I)** and **(II)** show bond lengths of N1–H1 (1.036 Å) and H1···O15 (1.634 Å) in **(I)**, and N1–H1 (1.036 Å), H1···O15 (1.682 Å), and H5···O18 (2.167 Å) in **(II)**, which coincide with the experimental X-ray crystallographic data. In both **(I)** and **(II)**, the N1–H1 bonds are shorter than the H–O bonds, and the presence of proton transfer validates their molecular salt nature (Fig. 4**)**.

### Hirshfeld surface analysis

**Fig. 5:**
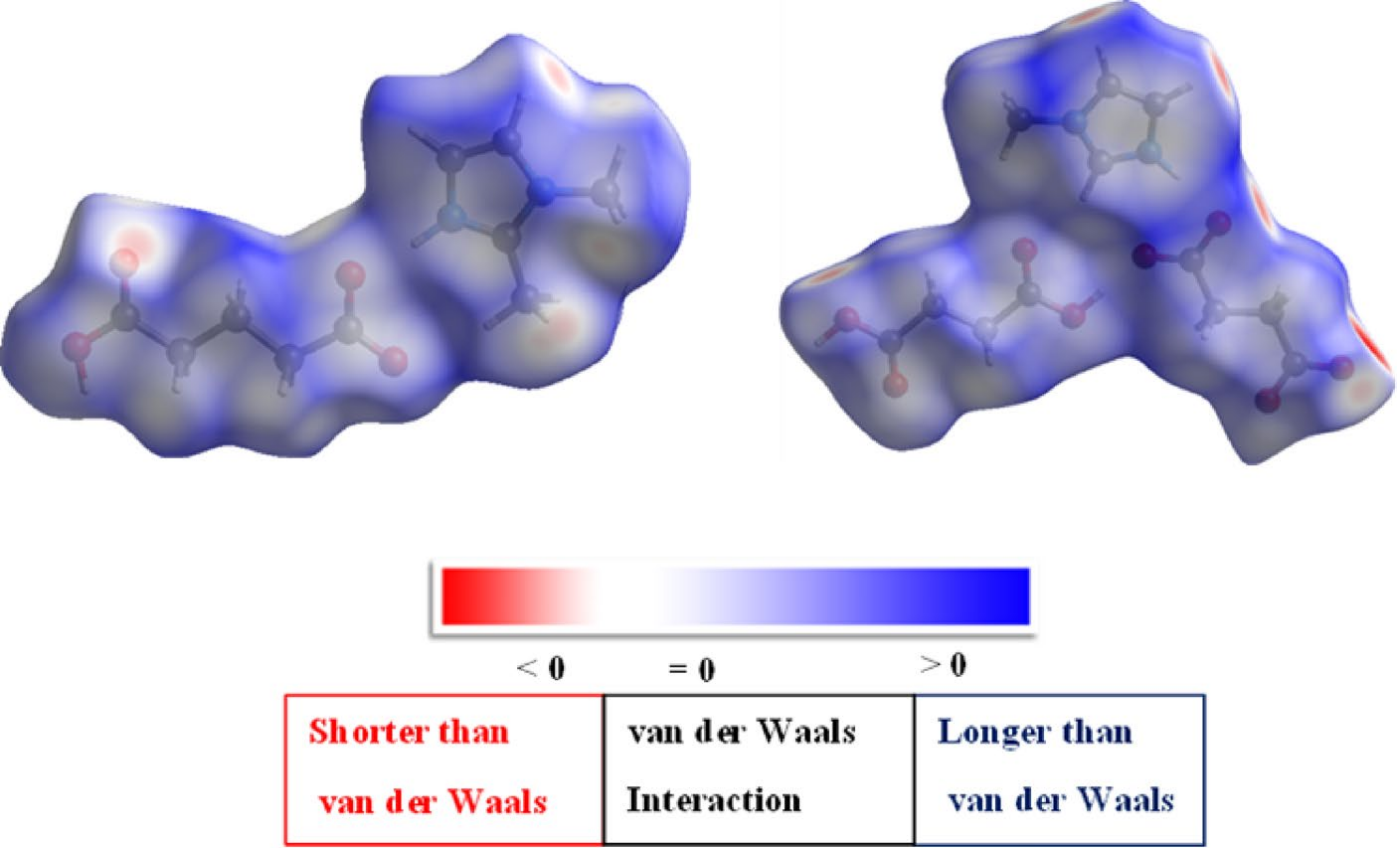
Hirshfeld d_norm_ surface maps of **(I) & (II)** for their intermolecular interactions.

Hirshfeld surface analysis is a useful tool for analyzing the electron density of a crystal into molecular fragments by characterizing the space that molecules occupy in the crystal^35^. It also gives more details about the intermolecular interactions of molecular crystals and plays a significant role in defining the surface characteristics of molecules. Hirshfeld surface investigations of (**I)** & (**II)** are performed by using the Crystal Explorer program from the determined X-ray crystallographic CIF file. Intermolecular interactions of these molecules are best quantified by using Hirshfeld surfaces and their corresponding two-dimensional fingerprint plots. The dnorm mapped surface of (**I)** & (**II)** are illustrated in **(**Fig. 5**)**. Positive dnorm values, shown in blue, imply contacts that are longer than the total of the van der Waals radii, whereas negative values, shown in red, suggest contacts that are shorter.

It can be seen that the length of the alkyl chain in organic acid-imidazole systems significantly impacts crystal packing and hydrogen-bonding networks by influencing intermolecular forces, steric hindrance, and molecular conformation^36^. From the Hirshfeld surface analysis, it was observed that as the alkyl chain increases, the number of van der Waals interactions between alkyl chains also increases. The results depict that the hydrogen bonding between the O-H…O and C-H…O atoms of both structures corresponds to the red patches on the surface map. Blue patches cover a wide area and are for the longer than van der Waals interactions, whereas white regions show the weaker van der Waals connection.

**Fig. 6:**
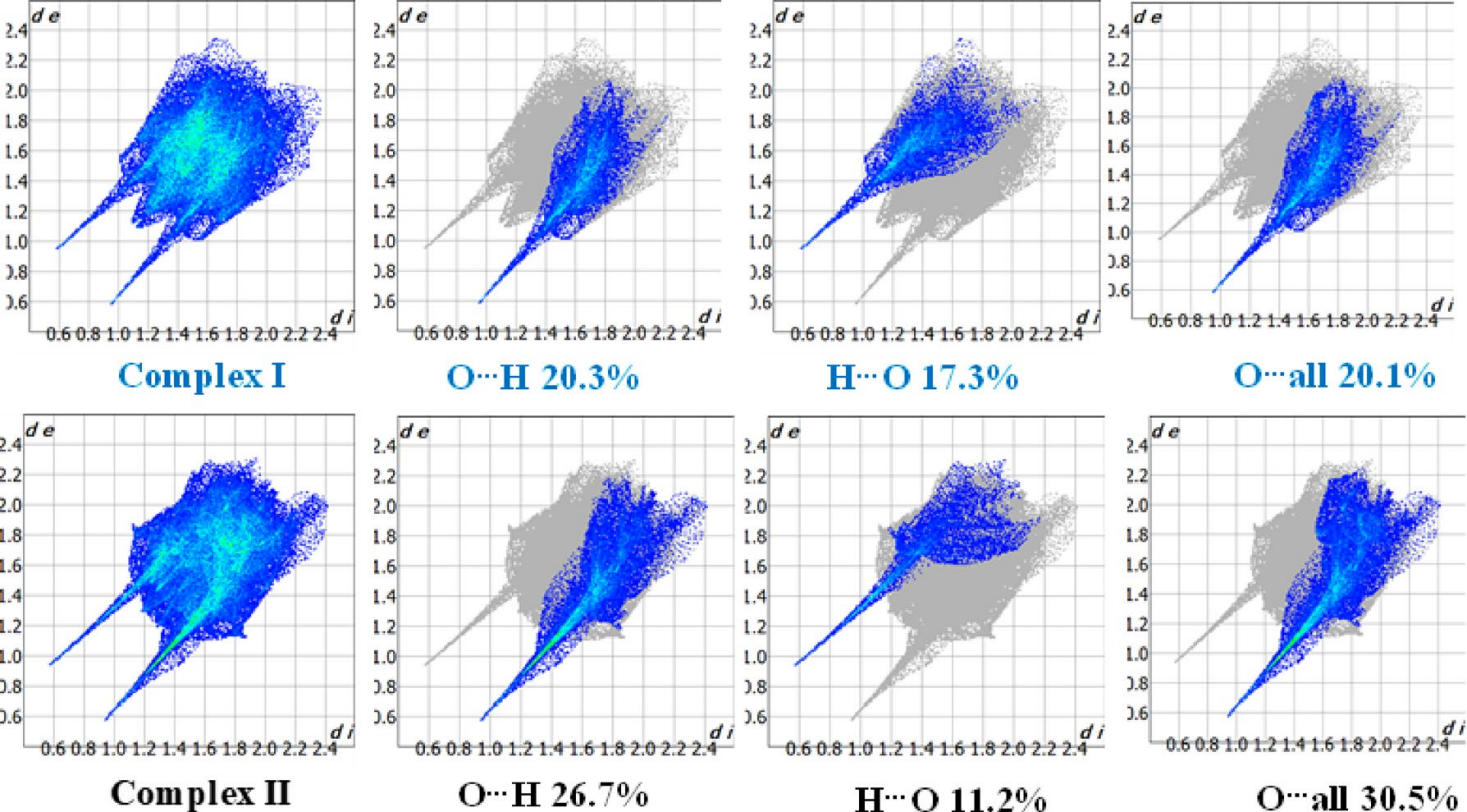
2D finger print Hirshfeld plots of (**I)** & (**II)** for their intermolecular contribution.

Additionally, 2D fingerprint plots for the intermolecular percentage contribution of atom to atom in both molecular salts are displayed in **(**Fig. 6**)**. The findings indicate that, for (**I)**, the O…H atom interaction contributes the largest intermolecular proportion, up to 20.3%, followed by O…all and H…O atom interactions, which account for 20.1% and 17.3% of the total, respectively. On the other hand, for (**II)**, the O…all atom interaction contributes the largest intermolecular proportion, up to 30.5%, followed by O…H and H…O atoms interactions, which account for 26.7% and 11.2% of the total, respectively.

### Interaction region indicator (IRI) analysis

The nature of intramolecular and intermolecular interactions of the studied structures can be better analyzed using the IRI approach^37^. Several interactions inside chemical systems can be simultaneously identified by the recently established real-space function, such as IRI. A constant pre-factor separates the IRI and RDG, which is essential to maintain equilibrium between covalent and non-covalent interactions. The IRI expression can be written as:

1 $$\:\mathrm{I}\mathrm{R}\mathrm{I}\:\left(\mathbf{r}\right)=\:\frac{\left|\nabla\:\rho\:\:\left(\mathbf{r}\right)\right|\:}{{\left[\rho\:\left(\mathbf{r}\right)\right]}^{\alpha\:}}$$

Here, $\:\rho\:$ is the electron density, $\:\mathbf{r}\:$is coordinate vector, whereas $\:\alpha\:$ is a variable parameter and it is an accepted value ($\:\alpha\:$ = 1.1). It is basically a scaled version of the gradient norm of electron density, whereas the RDG is a dimensionless form of electron density gradient. The RDG can be computed as follows:

2 $$\:RDG\left(\mathbf{r}\right)=\:\frac{1}{2\left({3\pi\:}^{2}\right){\:}^{1/3}}\frac{|\nabla\:\rho\:\left(\mathbf{r}\right)|}{\left[\rho\:\right(\mathbf{r}){]\:}^{4/3}}$$

**Fig. 7:**
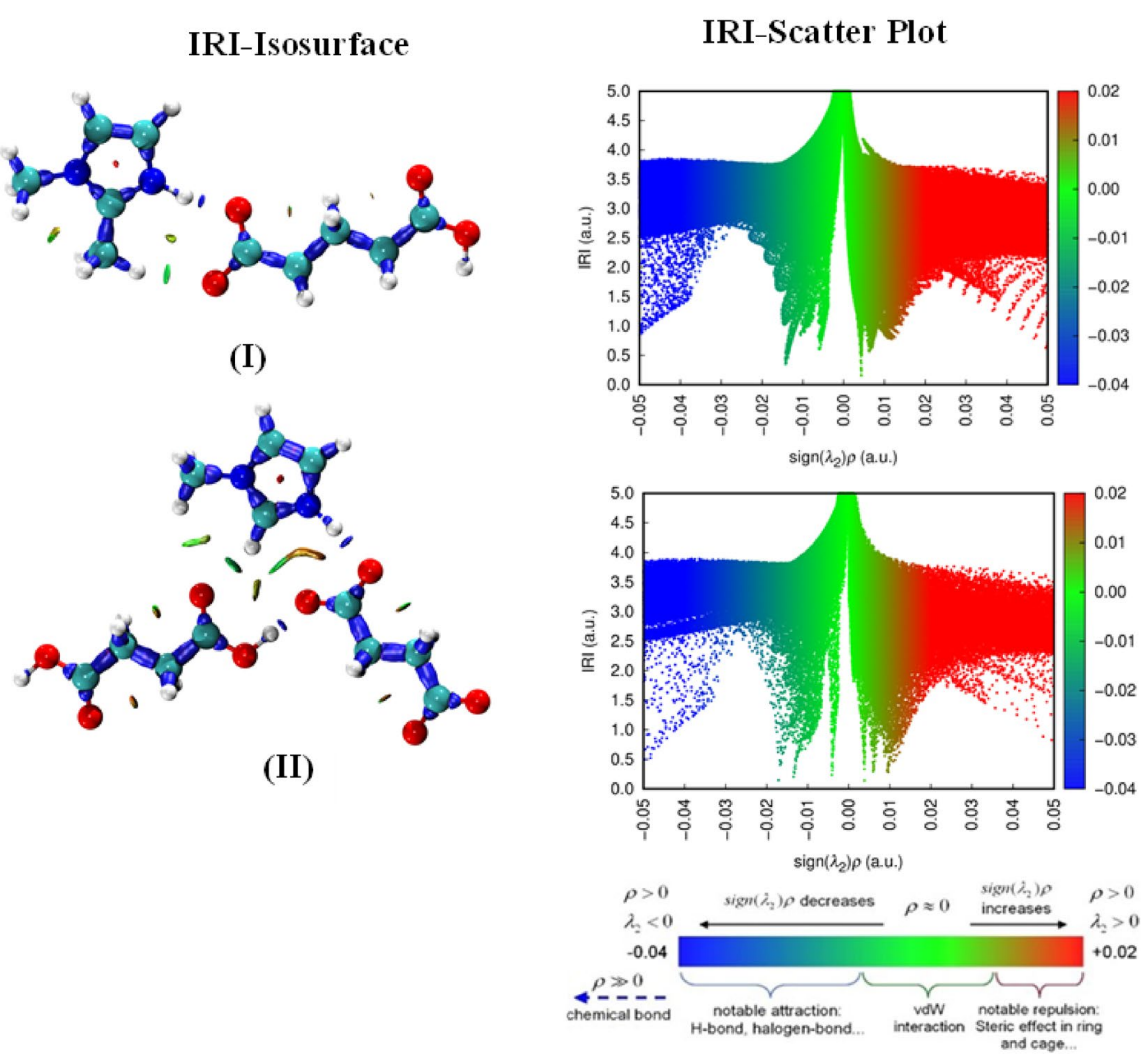
3D-isosurfaces and 2D-IRI scatter plots of **(I)** and **(II).**

The red, green, and blue patches on the 3D-IRI isosurfaces indicate repulsive, non-covalent, and attractive interactions. By projecting the sign (λ_2_)*ρ* function onto the 2D-IRI plots, it is possible to clearly illustrate the nature of the interaction regions. A region with a small sign (λ_2_)*ρ* ≈ 0 and low electron density shows no apparent interaction or the van der Waals (vdW) interaction, whereas, a region with a relatively large value of sign (λ_2_)*ρ* and high *ρ* indicates a reasonably strong interaction hydrogen & halogen bonding, etc.). Therefore, in order to understand the covalent and non-covalent interactions, the interpretation of 3D-IRI isosurfaces and 2D-IRI plots of structures has been carried out as illustrated in (Fig. 7).

The results demonstrate that these molecular salt exhibit extensive blue patches of hydrogen bonding and some green patches of weak dispersive vdW interactions. The blue patches on the **(I)** are more prominent than the **(II)**. Additionally, the presence of hydrogen bondings is supported by the appearance of distinct blue spikes ranging from − 0.03 to -0.05 au, in the 2D-IRI plots of the salts. The molecualr salt **(II)** exhibits low gradient, low density, and sharp spikes, whereas the spikes of **(I)** are less dense.

### Quantum theory of atoms in molecules (QTAIM) analysis

**Fig. 8:**
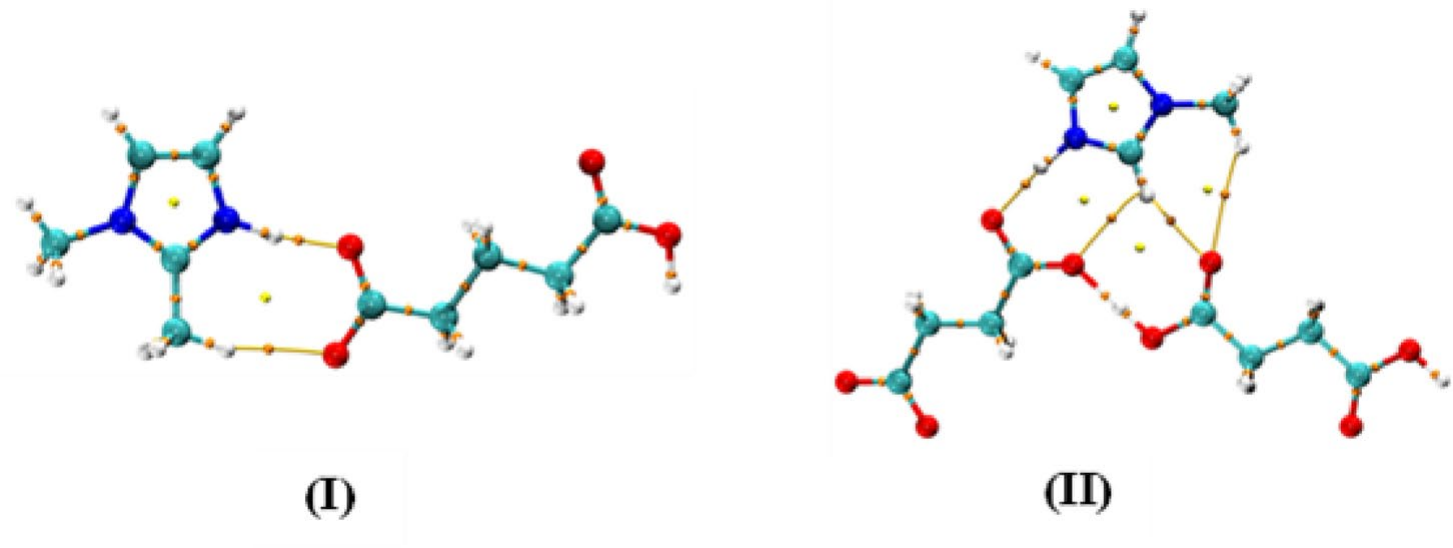
Pictorial representation of QTAIM of **(I)** & **(II)** (along BCPs as orange dots).

It is a topological tool utilized to examine the type and strength of different interactions based the electron density and various other parameters at the bond critical points^38^. It offers an elegant approach for identifying the nature of a bond based on its critical points, including the total electron density (*ρ*(r), Laplacian ∇^2^*ρ*(r), kinetic V(r) and potential G(r) electron energy densities and total energy density (H). An important factor influencing the strength of interactions is the electron density (*ρ*(r)). A negative Laplacian ∇^2^*ρ* (r), H(r) < 0, *ρ*(r) > 0.1 au and -G(r)/V(r) < 1 show strong bondings such as electrostatic interactions and H-bonding. Conversely, partial covalent and partial electrostatic forces only have H(r) < 0 with a positive Laplacian ∇^2^*ρ* (r), while weak interactions like van der Waals are demonstrated by a positive Laplacian ∇^2^*ρ* (r), *ρ*(r) < 0.1 au, H(r) > 0, and -G(r)/V(r) > 1. The topological diagrams with the corresponding BCPs of **(I)** & (**II)** are displayed in (Fig. 8**)** and their AIM topological parameters are given in (Table 3**)**.

**Table 3:** The AIM topological parameters as electron density (*ρ*), laplacian of electron density (∇^2^ρ), and the ratio of the kinetic electron density G(r) to potential electron density V(r) at BCPs of the structures (All the values are in a.u.)

Structures	BCP No.	Interaction	ρ	∇^2^ρ	G(*r*)	V(*r*)	H(*r*)	-G(*r*)/V(*r*)
**(I)**	59	N_imi_-H_imi_	0.3061	-0.0164	0.0503	-0.5128	-0.4624	0.0981
	53	O_a_- H_imi_	0.0562	0.1307	0.0374	-0.0421	-0.0047	0.8883
	35	H_imi_-O_a_	0.0141	0.0357	0.0091	-0.0094	-0.0002	0.9681
**(II)**	73	N_imi_-H_imi_	0.3103	-1.1681	0.0465	-0.5132	-0-4666	0.0907
	76	NH_imi_-O_a_	0.0501	0.1231	0.0336	-0.0364	-0.0028	0.9231
	62	CH_imi_-O_a_	0.0134	0.0488	0.0108	-0.0094	-0.0013	1.1489
	56	CH_imi_-O_a_	0.0171	0.0465	0.0121	-0.0123	-0.0003	0.9837
	53	CH_imi_- O_a_	0.0041	0.0167	0.0032	-0.0023	0.0009	1.3913

The results indicate that the present salts exhibit different types of bond critical paths. The **(I)** shows three BCPs including two at H_imi_-O_a_ and one at N_imi_-H_imi_, whereas **(II)** has four BCPs at the H_imi_-O_a_ and one at N_imi_-H_imi_ bond. Moreover, proton transfer is confirmed by the topological parameters, as indicated by the negative ∇^2^*ρ* (r), H(r) < 0, *ρ*(r) > 0.1 au and -G(r)/V(r) < 1 for the N_imi_-H_imi_ bonds with strong bonding interactions, including electrostatic contributions and hydrogen bonding.

### Frontier molecular orbital (FMO) analysis

**Fig. 9:**
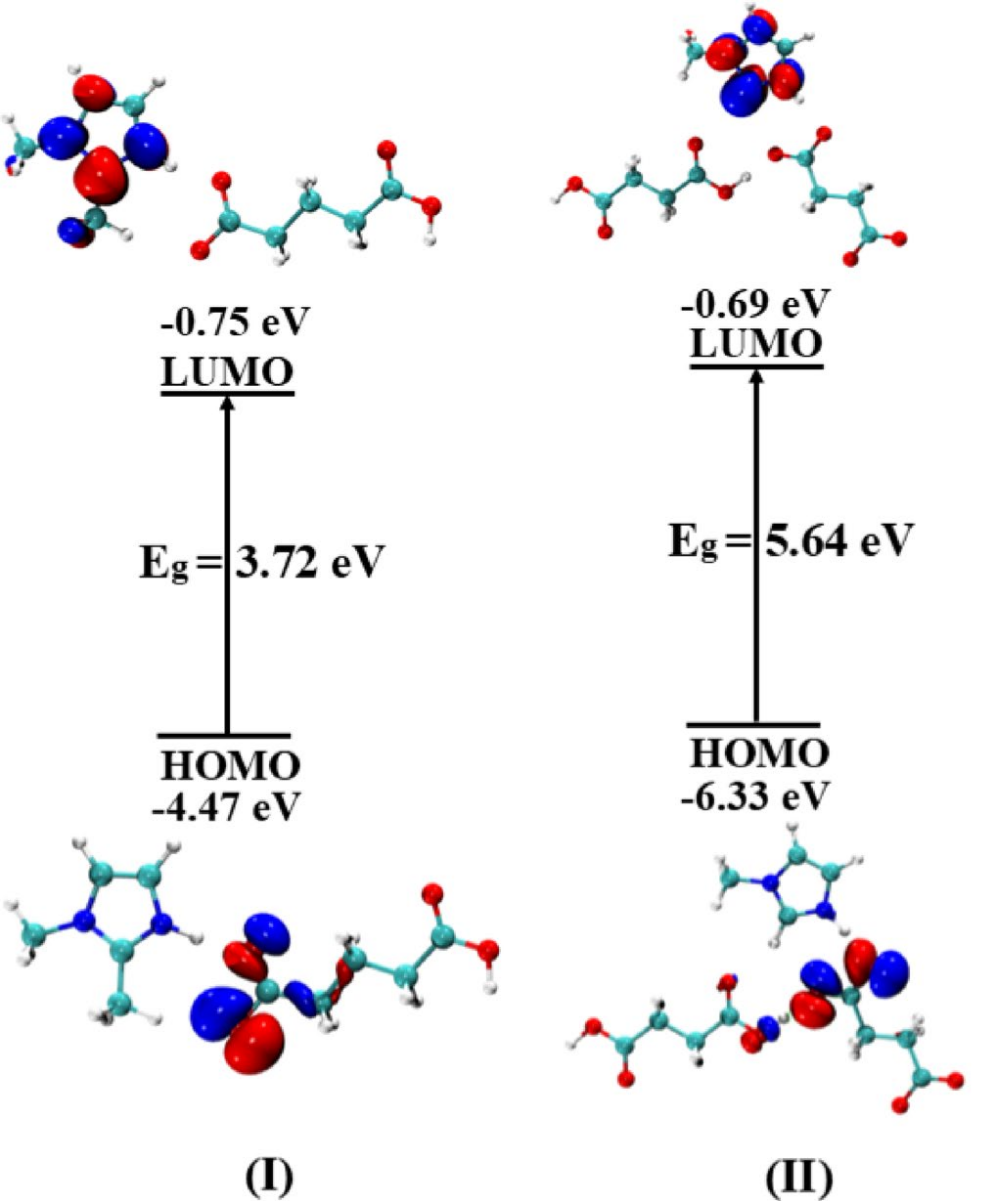
Frontier molecular orbitals surface plots of molecular salts and their energy gaps E_g_ (eV).

The FMO analysis can be used to examine the reactivity and electronic properties of any system^39^. The visual representation of HOMO and LUMO electron densities along with their energy gaps (E_g_) are represented in **(**Fig. 9**)**.

The results depict that HOMO in **(I)** is mainly localized on the acid, and the LUMO electron density is dispersed on its imidazole counterpart. In contrast, HOMO electron density in **(II)** is primarily located on the succinate ion, in conjugation with the imidazole moiety, while the LUMO density is concentrated on the imidazole ring. The HOMO-LUMO energy gaps of **(I)** and **(II)** are 3.72 eV and 5.64 eV, respectively. This distribution of electron density not only explains the differences in their energy gaps but also provides a clear insight into the relative stabilities of the salts. In **(I)**, the HOMOs electron density shows greater delocalization over the carboxylate group of counteracid, suggesting enhanced conjugation. This observation is consistent with its higher HOMO energy value (of -4.47 eV) and its lower E_g_ of 3.72 eV, indicating reduced kinetic stability and and increased reactivity. Conversely, in **(II)**, the HOMO electron density is more likely to be delocalized and primarily concentrated on the acid groups and results in a higher HOMO energy value (-6.33 eV). This distribution of electron density might be the reason for an increased E_g_ up to 5.64 eV, implying better kinetic stability.

## Conclusion

Crystals of 1-methylimidazole and 1,2-dimethylimidazole with glutaric and succinic acids are reported. Although the crystal structure of 1,2-dimethylimidazole with glutaric acid **(I)** has been previously described, the molecular salt of 1-methylimidazole with succinic acid **(II)** is characterized here for the first time. In both salts **(I)** and **(II)**, the crystal packing is stabilized by intermolecular N—H···O and C—H···O hydrogen bonds, confirming their molecular salt nature. The Hirshfeld surface analysis result indicates that, for **(I)**, the O…H atom interaction contributes the largest intermolecular proportion, up to 20.3%, followed by O…all and H…O atom interactions. While for **(II)**, the O…all atom interaction contributes the largest intermolecular proportion, up to 30.5%, followed by O…H and H…O atom interactions. On the other hand, computational methods confirm the hydrogen bond between the –NH groups in 1-methylimidazole and 1,2 dimethylimidazole. The interaction region indicator (IRI) approach has been used to analyze better the nature of intramolecular and intermolecular interactions of the studied salts, the results demonstrate that these salts exhibit extensive blue patches of hydrogen bonding and some green patches of weak dispersive vdW interactions. Meanwhile, the quantum theory of atoms in molecules (QTAIM) analysis was used to examine the type and strength of different interactions based the electron density and various other parameters at the bond critical points. The results indicate that the present salts exhibit different types of bond critical paths. Salt (I) shows three BCPs including two at H_imi_-O_a_ and one at N_imi_-H_imi_, whereas salt **(II)** has four BCPs at the H_imi_-O_a_ and one at N_imi_-H_imi_ bond. Also, the frontier molecular orbital (FMO) analysis has been used to examine the reactivity and electronic properties of the system, the results depict that HOMO in **(I)** is mainly localized on the acid part, and the LUMO electron density is dispersed on the imidazole ring. In contrast, HOMO electron density in **(II)** is primarily located on the succinate ion, in conjugation with the imidazole moiety, while the LUMO density is concentrated on the imidazole ring with better kinetic stability.

## Supplementary Information

Below is the link to the electronic supplementary material.

Supplementary Material 1

Supplementary Material 2

## Data Availability

Additional data analysed during this study have been deposited in the Cambridge Crystallographic Data Centre (CCDC): IDs 2408255-2408256 and also available as Supplementary material.
